# Facile synthesis of silver doped manganese oxide nanocomposite with superior photocatalytic and antimicrobial activity under visible spectrum

**DOI:** 10.1038/s41598-024-65749-z

**Published:** 2024-07-08

**Authors:** Sherif Elbasuney, Ahmed M. El-Khawaga, Mohamed A. Elsayed, Amir Elsaidy, M. Yehia, Miguel A. Correa-Duarte

**Affiliations:** 1 Head of Nanotechnology Research Center, Military Technical College, Cairo, Egypt; 2 Department of Basic Medical Sciences, Faculty of Medicine, Galala University, New Galala City, 43511 Suez Egypt; 3 Head of School of Chemical Engineering, Military Technical College, Cairo, Egypt; 4 School of Chemical Engineering, Military Technical College, Cairo, Egypt; 5 Biomedical Research Center (CINBIO), and Institute of Biomedical Research of Ourense-Pontevedra-Vigo (IBI), Universidad de Vigo, 36310 Vigo, Spain

**Keywords:** Nanoparticles, Photocatalysis, Water-treatment, Antimicrobial activity, Nanoscience and technology, Nanobiotechnology, Nanostructures, Environmental chemistry

## Abstract

Water pollution and antimicrobial resistance (AMR) have become two global threats; 80% of diseases and 50% of child deaths are due to poor water quality. In this study, hydrothermal processing was employed to manufacture manganese oxide nanorods. Silver dopant was deposited on the surface of manganese oxide. XRD diffractogram confirmed the facile synthesis of Ag/Mn_2_O_3_ nanocomposite. XPS survey analysis demonstrated silver content of 9.43 atom %. Photocatalytic measurements demonstrated the outstanding efficiency of the Ag-Mn_2_O_3_ compared to virgin oxide particles under visible radiation. Degradation efficiencies Mn_2_O_3_ and Ag/Mn_2_O_3_ on methyl orange (MO) dye was found to be 53% and 85% under visible spectrum. Silver dopant was found to decrease the binding energy of valence electrons; this action could support electron–hole pair generation under visible spectrum and could promote catalytic performance. Ag/Mn_2_O_3_ NPs demonstrated most effective performance (95% removal efficiency) at pH 3; this could be ascribed to the electrostatic attraction between positively charged catalyst and the negatively charged MO. Ag/Mn_2_O_3_ demonstrated enhanced antibacterial activity against Gram-positive *Staphylococcus aureus* (*S. aureus*) (19 mm ZOI), and Gram-negative *Escherichia* *coli* (*E. coli*) (22 mm ZOI) respectively; the developed nanocomposite demonstrated advanced anti-film activity with inhibition percentage of 95.5% against *E. coli* followed by 89.5% against *S. aureus*.

## Introduction

The global scale of water purification has become an urgent concern due to modern industries and population growth. Recently, water pollution and antimicrobial resistance (AMR) have become two global threats^1,2^. It has been reported that 80% of diseases and 50% of child deaths are due to poor water quality^3^. Approximately 17–20% of global pollutants can be attributed to the textile industry, according to the World Bank estimates^4^. The persistent nature of these pollutants stems from the stability of water-soluble Azo dyes; that would result in prolonged environmental impacts^5,6^. Methyl Orange (MO) belongs to the azo dye family; it is known to be carcinogenic organic substance. MO is widely-used in paper manufacturing, pharmaceutical, food industries, and in research laboratories as an acid–base indicator^7,8^. Direct contact with the eyes can result in chemical burns. When MO comes in contact with the skin, it may produce toxic effects. There is a significant interest towards advanced water treatment strategies, due to the extensive contamination of water resources^9^. The provision of un-contaminated water and the development of sustainable water purification methodologies are of paramount importance from industrial, societal, and environmental perspectives. The integrity of the natural ecosystem is being critically compromised due to the accelerated speed of global industrialization. Industrial discharge of hazardous dyes exposes detrimental effects on the environment. Conventional treatment methods that rely on centralized systems are no longer sustainable.

In recent years, the problem of bacteria antibiotic resistance to traditional antibiotics has reached a critical level^10^. The world population would be facing a real possibility of post-antibiotic era, where common infections and minor injuries could become life-threatening. According to World Health Organization, anti-microbial resistance, is expected to withstand the death of one person every three seconds, by 2050^11^. Traditional approaches to water treatment like coagulation, flocculation^12^, sedimentation^13^, and filtration^14^, have been employed. However, these methods often fall short in terms of efficiency, cost-effectiveness, and environmental impact. The utilization of nanomaterials in photocatalytic processes for treatment of harmful textile dyes has been identified as an environmentally friendly approach. The distinctive characteristics of photocatalytic nanomaterials experienced a significant impact on textile dye treatment^15^. Nanotechnology has been applied as a prominent approach for waste-water treatment^16^. NPs experienced superior physical and biological reactivity owing to their high interfacial surface area. Enhanced adsorption and photocatalytic activities of NPs could secure new sustainable solution for waste-water treatment. Semiconductors are of interest due to low cost, high physical and chemical stability, with minimum toxicity. However, the wide band-gap energy limits the photocatalytic applications to only 5% of the entire solar spectrum. Doping with rare earth elements was found to enhance the photocatalytic activities of semiconductors. Therefore, the NP band-gap value can be adjusted by controlling the size without altering their chemical composition. Narrow band-gap matching with the visible light absorption is highly appreciated for advanced photocatalyst. Extensive research has been conducted on metal oxides to enhance their functionality and to investigate their exceptional physical properties^17–19^. Many metal-based catalysts are only active under ultraviolet irradiation, which accounts for just 5% of solar radiation; this action could impose the use of high-energy consumption Xenon or UV lamps. Manganese oxides (Mn_y_O_x_) can offer different oxidation states; manganese oxide has been investigated as effective photocatalyst for organic pollutants degradation. Manganese oxide is favored for simple production processes, low cost, low toxicity, high adsorption, and oxidation capabilities. Various studies have been conducted on Mn_y_O_x_-based photocatalysts to remove aquatic pollutants due to their large specific surface area and excellent adsorption properties^20,21^. Silver in its free ionic form is highly toxic to human cells. It has been demonstrated that doping with silver on metal oxide eliminated silver ion toxicity to human cells. Additionally, doping with silver can inherit metal oxide particles superior antibacterial activity^22^.

Manganese oxide with consistent product quality has been developed; doping with silver on oxide lattice could be useful. It has been reported that doped manganese oxides materials experienced good catalytic activity^23^. This study reports on the facie synthesis of colloidal manganese oxide nanorods via hydrothermal synthesis. The potential impact of silver dopant on adsorption capabilities, and photocatalytic activity was assessed to virgin manganese oxide. The potential impact of silver on antimicrobial and anti-biofilm activities against some pathogenic microbes was assessed to be virgin manganese oxide. This study shaded light on the facile development of narrative cost effective, photocatalyst for wastewater treatment.

## Materials and methods

### Chemicals

Manganese nitrate (99%, Aldrich) was employed as the precursor for Mn_2_O_3_ synthesis. Hydrogen peroxide (30 V %, Aldrich) was employed as a source for OH^−^ ion. Methyl Orange (MO) obtained from Aldrich (Aldrich, purity <  = 100%), was employed as a contaminant. Ultrapure milli-Q water was employed as the water source. Silver nitrate (99%, Aldrich) was employed as a precursor for silver deposition. All chemicals were utilized without undergoing additional purification procedures.

### Manganese oxide synthesis

Manganese oxide nanorods were fabricated in a continuous manner via hydrothermal processing. Manganese metal salt flow (B) was immediately mixed with supercritical water (ScW) flow (A). Manganese oxide nanorods were continuously produced in the reactor (R) at the boundary of the two fluids. Flow (A) was 20 ml/min super-critical water at 400  ^0^C and 240 bars. Flow (B) was 0.05 M manganese nitrate at 10 ml/min, 25  ^0^C and 240 bars (Supplementary Figure S1). Further details about the hydrothermal processing of various forms of manganese-oxides can be found in the following reference^24,25^.

### Ag/Mn_2_*O*_3_ synthesis

Manganese oxide particles were dispersed in silver nitrate solution using an ultrasonic bath for 1 h. The weight percentage of Mn_2_O_3_ to silver ion was 9:1. Subsequently, silver deposition was conducted under strong magnetic stirring using sodium borohydride; the stoichiometric ratio of sodium borohydride to silver ion was 3:1. This approach could secure optimal homogeneity and even silver deposition on Mn_2_O_3_ surface.

### Characterization of catalyst NPs

Transmission Electron Microscope (TEM) (JEM-HR-2100, Joel Corporation) and Scan Electron Microscope (SEM) (Zeiss EVO-10, Carl Zeiss Corporation) were used to study the morphological structure of manufactured Mn_2_O_3_ nanorods. The crystalline phase of manganese oxide was examined using XRD D8 advanced by Burker Corporation. The elemental composition of silver doped manganese oxide, and binding energy of valence electron were examined using an X-ray photoelectron spectroscopy XPS/ESCA equipment.

### Photocatalytic performance of Ag/Mn_2_O_3_ against methyl orange

A nanocomposite catalyst (10 mg) was introduced into 50 ml aqueous solution containing methyl orange (MO), with an initial concentration (C_0_) of 10 mg/L. The mixture was stirred continuously at room temperature (25 °C) for 30 min in the absence of light to establish equilibrium between adsorption and desorption processes. Subsequently, a simulated UV light source in the form of UV lamp was employed to illuminate the solution containing the photocatalyst and MO. The UV lamp was positioned axially within a quartz immersion tube. At fixed time intervals, a syringe equipped with a filter (pore size of 0.22 μm) was employed to extract a 1 ml sample of the MO suspension. The degradation rate of MO was determined by assessing the change in MO concentration over the course of irradiation time using a UV–visible spectrophotometer (Agilent Technologies Cary 60 UV–visible) at a wavelength (λmax) of 464 nm. Deionized water served as the reference medium^26^.

### Antimicrobial activity and minimal inhibitory concentration (MIC)

The antimicrobial efficacy of the virgin Mn_2_O_3_ and Ag/Mn_2_O_3_ was assessed using the agar-disc diffusion method^27^ against both Gram-negative (*Escherichia coli ATCC 25922*), and Gram-positive (*Staphylococcus aureus ATCC 25923*) bacteria. For performance comparison, conventional antibiotic discs containing gentamycin (CN) at a concentration of 10 μg/disc and with a diameter of 6.0 mm were employed alongside the nanoparticle under investigation.

The minimum inhibitory concentrations (MIC) of the potent antimicrobial samples were determined using the serial dilution technique within a Luria–Bertani (LB) medium^28^. This procedure involved the utilization of negative controls, which included the medium broth, as well as positive controls represented by the target pathogenic microorganisms along with the medium broth. The synthesized nanoparticle (initial concentration from 20.0 μg/ml to 0.625 μg/ml) were included in the assessment. Following that, the inoculum of the examined bacteria and yeast was set to a concentration of 0.5 McFarland. The MIC values were ascertained after a 24 h incubation period at a temperature of 36.0 ± 1.0 °C^29^. MIC was defined by using the ELISA plate method after setting the fixed wavelength at 600 nm^30^. The MIC was described as the lowest concentration of the virgin Mn_2_O_3_ and Ag/Mn_2_O_3_ that inhibits 99.0% of the growth of the tested bacteria.

### Antibiofilm activity of synthesized nanoparticles

Qualitative assessment regarding the inhibition of biofilm formation was conducted, via the methodology outlined by Christensen et al.^31^. Comprehensive examination of biofilm formation was carried out within the inner surface of test tubes, both in the absence and presence of Ag/Mn_2_O_3_ NPs. The anti-biofilm properties of Ag/Mn_2_O_3_ NPs at a concentration of 10.0 μg/mL were assessed and compared to a control group (untreated). Nutrient broth medium (5 mL) was introduced into all tubes, followed by the inoculation of the test bacteria. That was adjusted to a concentration of 1–3.5 × 10^8^ colony-forming units per milliliter (CFU/mL) equivalent to 0.5 McFarland standardization. Subsequently, they were incubated at 37.0 ± 0.5 °C for 24 h. The media from both the control and treated tubes were discharged; subsequently the tube was filled with phosphate buffer saline (PBS) at pH 7.0. The bacterial cells adhered to the tube walls were dislodged by treating with 5 mL of 3.5% sodium acetate for approximately 20 min, followed by a thorough cleaning with de-ionized water. The biofilms formed inside the tubes were stained with 20 mL of 0.15% Crystal Violet (CV) and subsequently rinsed with de-ionized water to remove excess CV. It is noteworthy that for semi-quantitative assessment of antibiofilm activity, 5 mL of absolute ethanol was introduced to dissolve the stained bacterial biofilms^32^. The optical density (O.D.) of the stained bacterial biofilms was measured using a UV–Vis spectrophotometer at 570.0 nm^33^. The inhibition percentage of bacterial biofilm formation was calculated using Eq. 1^34^:

1 $$ Biofil{\text{m}} \;inhibition\% = \left[ {\left( {O.D.Control\;sample - O.D.treated\;sample} \right)/O.D.Control\;sample} \right] \times 100 $$

## Results and discussion

### Characterization of nanocatalyst

**Figure 1:**
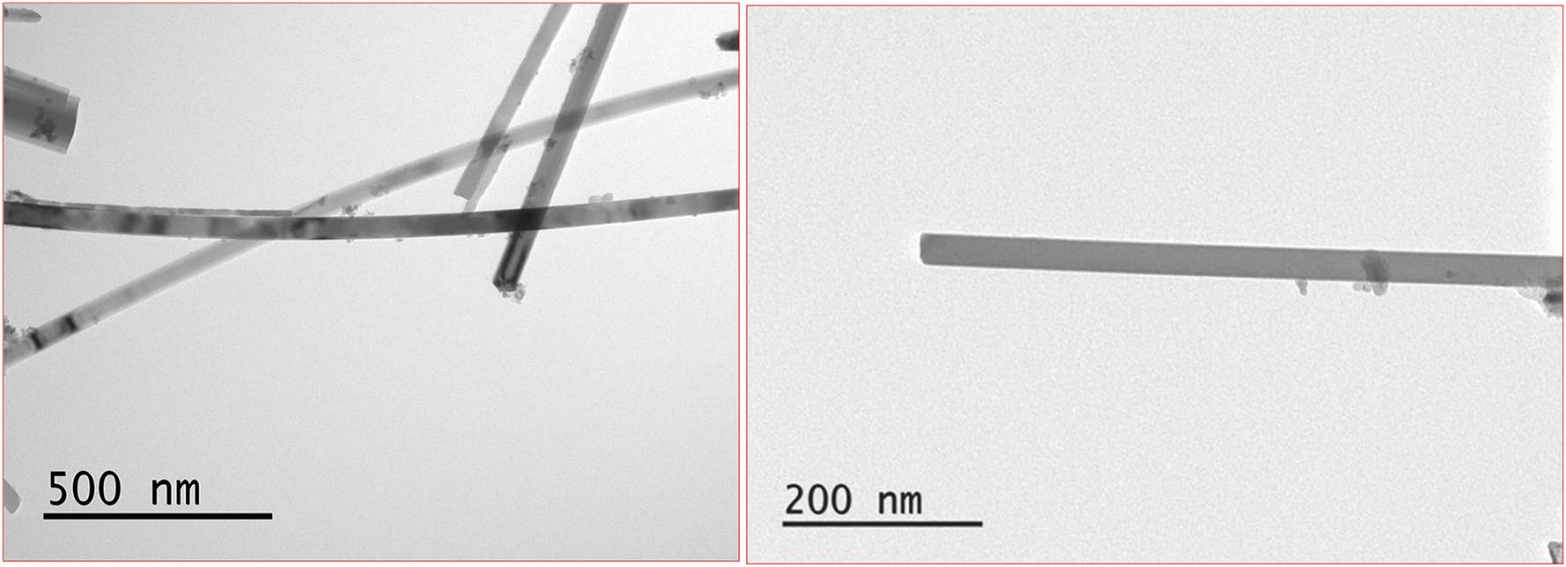
TEM micrographs of Ag/Mn_2_O_3_ nanorods.

Ag/Mn_2_O_3_ demonstrated nanorods of 500 nm length and 20 nm diameters. High quality particles were assessed via TEM micrographs as represented in Fig. 1.

**Figure 2:**
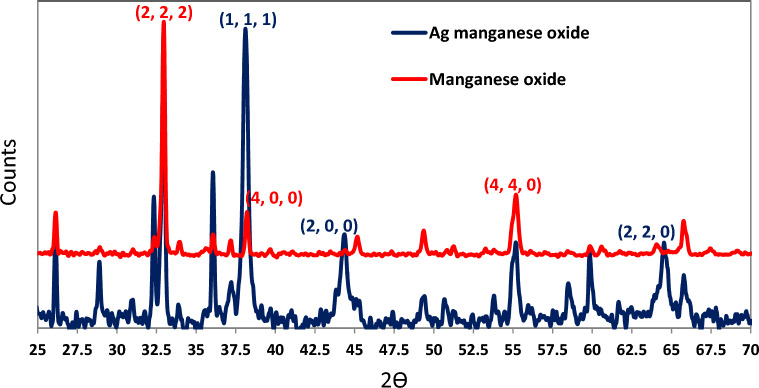
XRD of synthesized Ag/Mn_2_O_3_.

XRD diffractogram of synthesized Mn_2_O_3_ demonstrated crystalline structure with 6 sharp peaks at 2Ɵ = 32.95, 38.24, 45.18, 49.35, 55.19, 65.81 corresponding to (222), (400), (323), (431), (440), (622) planes respectively. XRD diffractogram of synthesized Ag/Mn_2_O_3_ confirmed the proper deposition of silver nanocatalyst on the surface of Mn_2_O_3_. Silver particles demonstrated characteristic sharp peaks at 2Ɵ = 38.11, 44.27, 64.42, 77.47 corresponding to (1,1,1), (2,0,0), (2,2,0), and (3,1,1) respectively (Fig. 2).

**Figure 3:**
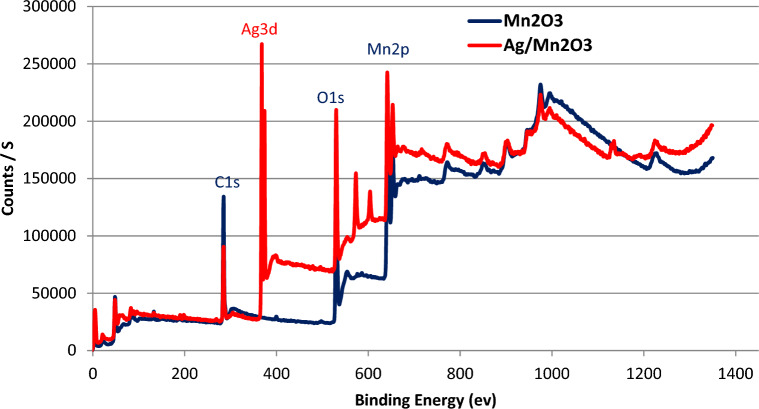
Survey of elemental composition of Ag/Mn_2_O_3_ to virgin Mn_2_O_3_.

Average crystallite size of Mn_2_O_3_ NPs was further evaluated using Debye-Scherer Eq. (2) via the major diffraction peak^35^.

2 $$ D\; = \;\frac{K\lambda }{{\beta \;{\text{COS}}\theta }} $$

where k is a constant equal to 0.94, λ is the wavelength, β is the full width at half maximum height (FWHM) of the diffraction peak in radians, and θ is the Bragg angles of the main planes. Crystallite size evaluation was found to be in good agreement with TEM micrographs. SEM micrographs demonstrated a great tendency to aggregate on drying. Elemental mapping confirmed the uniform dispersion of silver on the surface of Mn_2_O_3_ NPs (Figure S2). Elemental composition was investigated using XPS analysis. Atomic ratios were computed from peak intensity. Survey composition of Ag/Mn_2_O_3_ was verified to virgin Mn_2_O_3_ is represented in Fig. 3.

Elemental survey confirmed the proper deposition of elemental silver element. Elemental composition of Ag/Mn_2_O_3_ was evaluated to virgin Mn_2_O_3_ (Table 1).

**Table 1:** Elemental composition of Ag/Mn_2_O_3_ to Mn_2_O_3_ using XPS spectroscopy.

XPS elemental atomic percentage (At. %)					
Sample	C1s	O1s	Mn_2_p	Others	Ag3d
Mn_2_O_3_	50.58	35.88	9.67	3.87	––
Ag/Mn_2_O_3_	39.24	37.43	11.65	2.25	9.43

**Figure 4:**
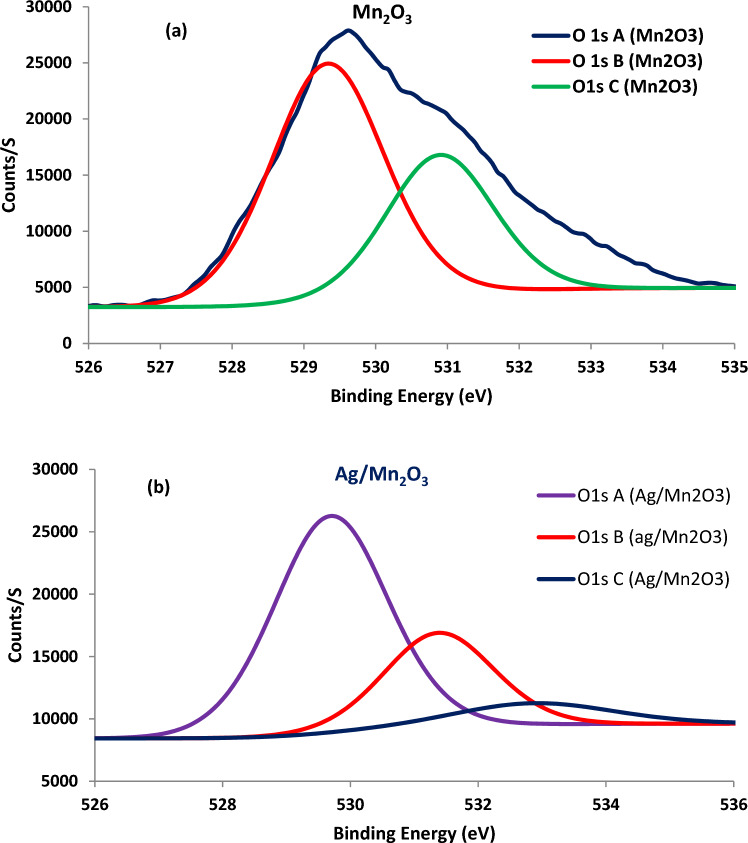
The impact of silver dopant on the binding energy of surface oxygen electrons (O1s).

Elemental composition confirmed the successful deposition of silver on the surface of Mn_2_O_3_ particles. Silver dopant was found to have a significant impact on binding energy (BE) of surface oxygen; silver dopant offered low binding energy of surface oxygen. Therefore, surface oxygen electrons could be involved more effectively into catalytic reactions. The impact of silver dopant on O1S binding energy is demonstrated in Fig. 4.

Silver dopant was found to enhance the content of O1s A, with low BE; this was accomplished on the extent of O1s B and C with high BE (Table 2).

**Table 2:** The impact of silver dopant on binding energy of O1 S.

Name	B.E	Mn_2_O_3_	Ag/Mn_2_O_3_
		Atomic %	Atomic %
O 1s A	529.33	55.14	*64.26*
O 1s B	530.9	30.64	26.89
O 1s C	532.5	14.22	*8.85*

**Figure 5:**
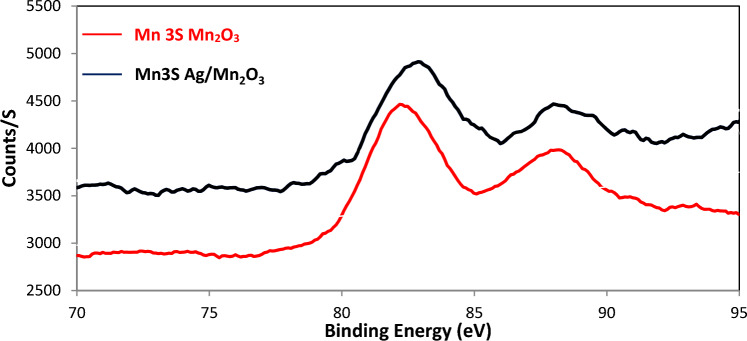
The impact of silver dopant on Mn 3S binding Energy.

Furthermore, silver dopant was found to decrease the binding energy of Mn 3S electrons, via the increase of Mn3s A (with low BE) on the extent on Mn3S B (with high BE) (Fig. 5).

Silver dopant was found to increase the atomic percent of Mn3 s A (with low BE) on the extent of Mn3 s B (with high BE) (Table 3).

**Table 3:** The impact of silver dopant on binding energy of O1 S.

Name	B.E	Mn_2_O_3_	Ag/Mn_2_O_3_
		Atomic %	Atomic %
Mn3 s A	82.8	64.68	*74.14*
Mn3 s B	88.34	35.32	*25.86*

**Figure 6:**
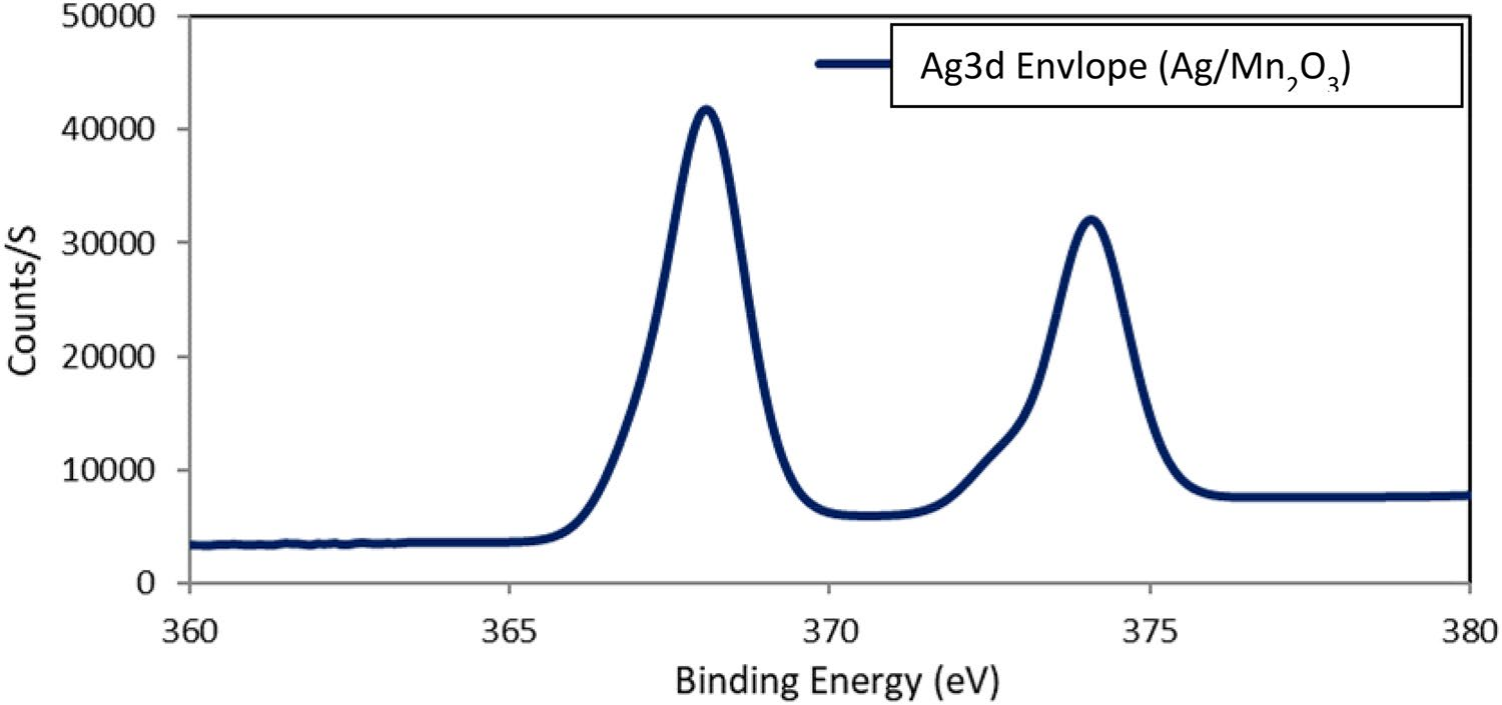
Binding Energy of Ag 3d silver dopant.

XPS spectrum of silver dopant confirmed the proper deposition of elemental silver. Ag 3d envelope is represented in Fig. 6.

It is obvious that elemental silver was successfully deposited on the surface of Mn_2_O_3_ particles.

### Photocatalytic performance of Ag/Mn_2_O_3_ NPs

**Figure 7:**
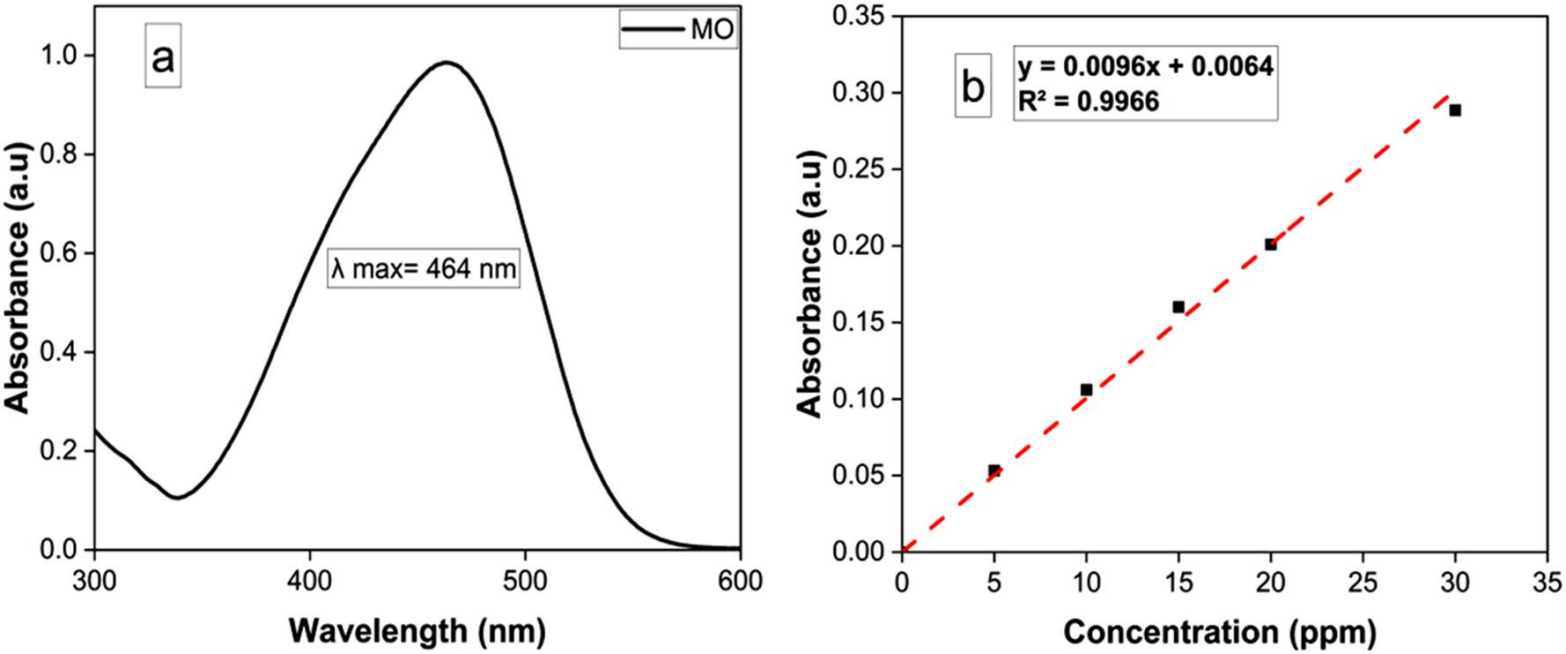
UV–Vis spectrum of MO (10 ppm) (**a**), Calibration curve of different concentrations of MO (**b**).

The removal of MO was monitored at optimum absorbance wavelength, λmax = 464 nm^36^. The UV–Vis spectrum of MO (10 ppm), and absorbance-concentration calibration curve is represented in Fig. 7.

**Figure 8:**
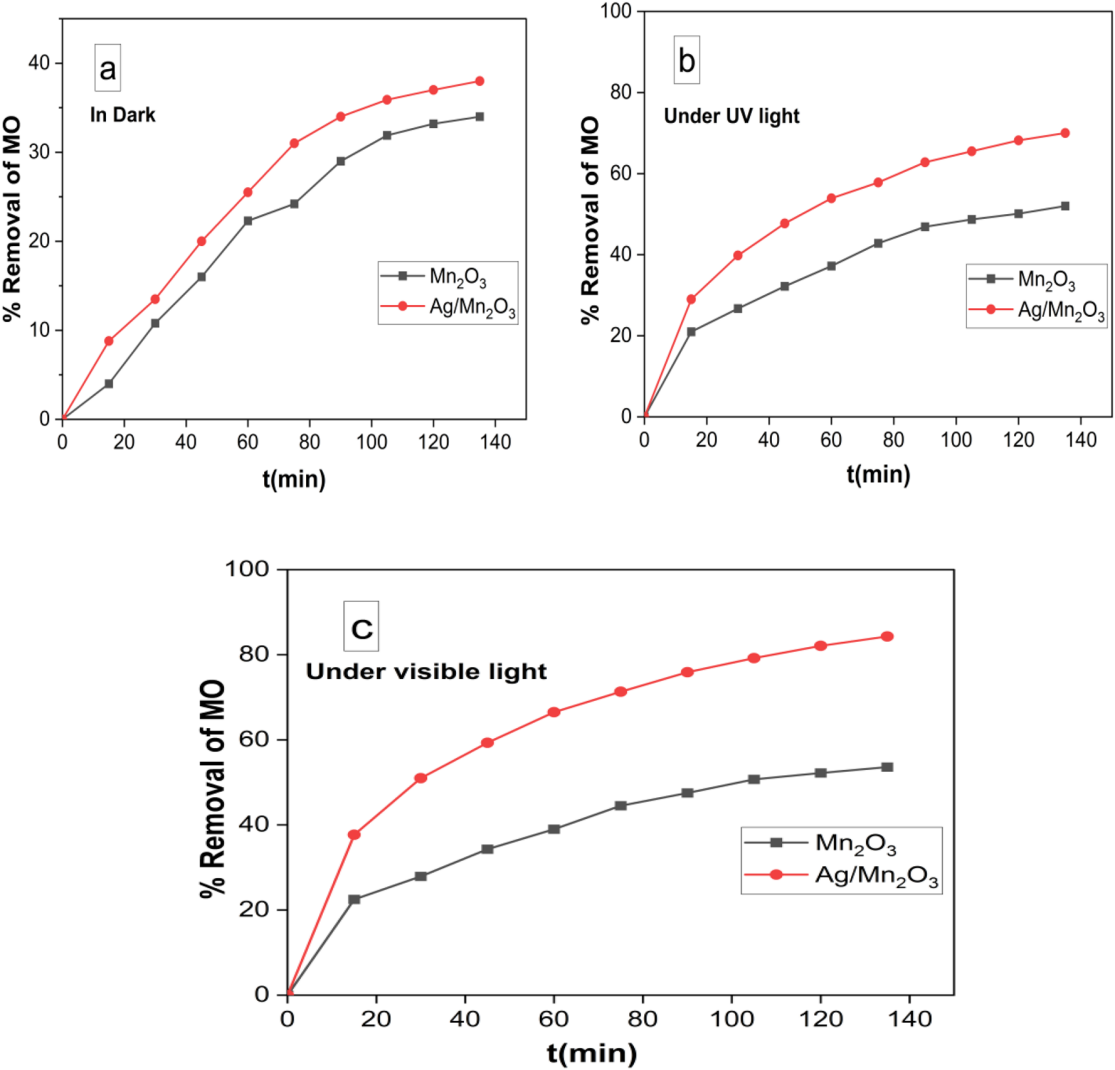
Removal % of MO via: adsorption in dark (**a**), Photocatalysis via UV irradiation (**b**), and photocatalysis under visible light irradiation (**c**).

The removal efficiency due to adsorption (in the absence of light) during the 135 min was approximately 34 and 38% for Mn_2_O_3_ and Ag/Mn_2_O_3_, respectively. On the other hand, the photocatalytic degradation of MO under UV light by Mn_2_O_3_ and Ag/Mn_2_O_3_ nanocatalyst reached 52 and 70%, respectively after 135 (Fig. 8b). The main finding in this study is that the photocatalytic performances of Ag/Mn_2_O_3_ nanocatalyst under visible light reached 85%, compared with 53% of Mn_2_O_3_ (Fig. 8c).

The superior photo-catalytic performance of Ag/Mn_2_O_3_ could be attributed to the presence of a metal–semiconductor hetero-junction within the nanocomposite; that could promote effective charge separation and light absorption.

#### Effect of pH on MO removal

**Figure 9:**
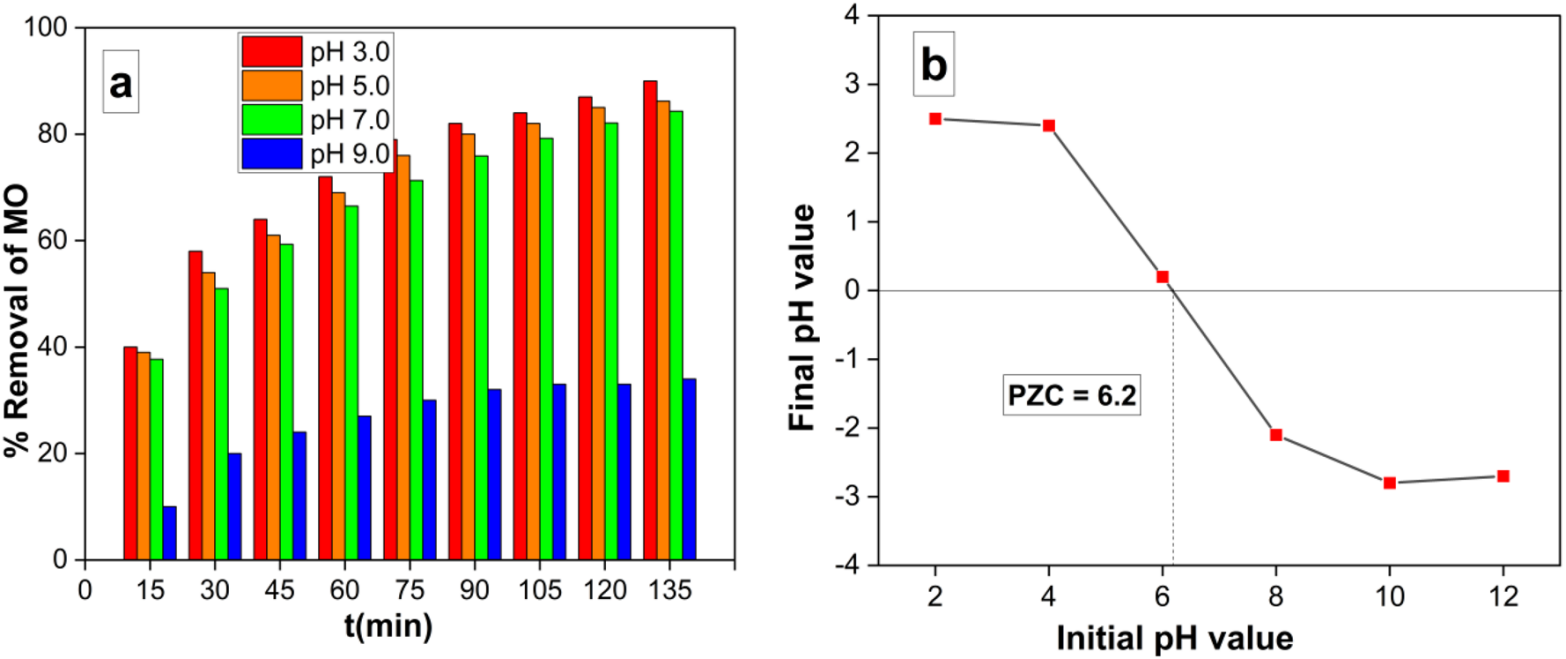
Showing the variation of MO removal (%) with time at different solution pH (3.0, 5.0, 7.0 and 9.0) (10 mg g of Ag/Mn_2_O_3_ NPs in 50 ml of 10 mg/l MO at 25 °C) (**a**), Point of zero charge (PZC) of Ag/Mn_2_O_3_ NPs at different pH values (**b**).

The dependence of removal efficiency on solution pH was assessed. The influence of initial pH values was conducted under specified conditions (10 mg of the prepared nanocomposite, 50 ml of a 10 mg/L MO solution, temperature maintained at 25 °C); the experiment time was 90 min. Graphical representation could assess the variation in MO removal (%) over time at different solution pH levels (3.0, 5.0, 7.0, and 9.0). The maximum MO removal at equilibrium was observed at pH 3.0 (Fig. 9a). The point of zero charge (PZC) of the Ag/Mn_2_O_3_ nanocomposite was determined; 0.01 g of Ag/Mn_2_O_3_ nanocomposite was introduced into 50 mL of a 0.01 M NaCl solution. The pH of the solutions was adjusted to achieve pH values of 3, 5, 7, and 9. Subsequently, the samples were stirred at 200 rpm for 48 h, and pH measurements were conducted after the magnetic separation of Ag/Mn_2_O_3_ NPs. The pH value corresponding to the Point of Zero Charge (PZC) was determined by constructing a plot illustrating the final pH against the initial pH (Fig. 9b). The PZC pH point was identified as the pH value where no substantial disparity existed between the final and initial pH values. When the solution’s pH matches the PZC pH, the surface charge of the photocatalyst becomes neutral; this could result in negligible electrostatic forces between the photocatalyst surface and ions (such as MO ions). The surface charge of the photocatalyst Ag/Mn_2_O_3_ becomes positive, when the pH is less than the PZC. The surface of photocatalyst becomes negative, when the pH exceeds the PZC.

The PZC pH for Ag/Mn_2_O_3_ NPs was calculated to be 6.2. This explains why the photocatalytic degradation of MO was most effective at pH 3.0 (Fig. 9a). At this pH, the net surface charge of Ag/Mn_2_O_3_ NPs is positive, resulting in attraction between the positively charged catalyst and the negatively charged MO; this action could offer enhanced photocatalytic efficiency.

#### Effect of initial concentration of MO

**Figure 10:**
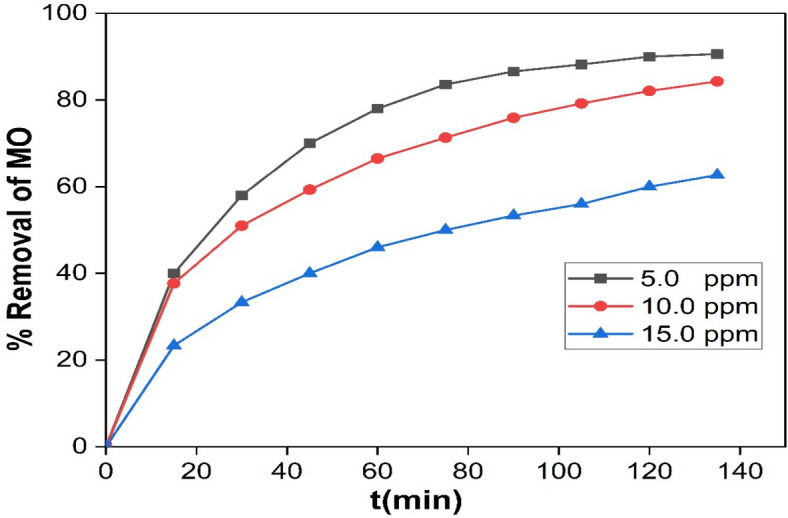
The variation of percent removal as a function of contact time at different initial MO concentration (5, 10 and 15 ppm) at pH 3.0 and 10.0 mg Ag/Mn_2_O_3_.

As the initial concentration of MO could play a pivotal role in the removal efficiency. The variation in removal percentage over time at different initial MO concentrations (5.0, 10.0, and 15.0 ppm) is represented in Fig. 10. The results revealed an inverse relationship between degradation efficiency with MO concentration.

#### Effect of the nanocomposite dose on degradation efficiency

**Figure 11:**
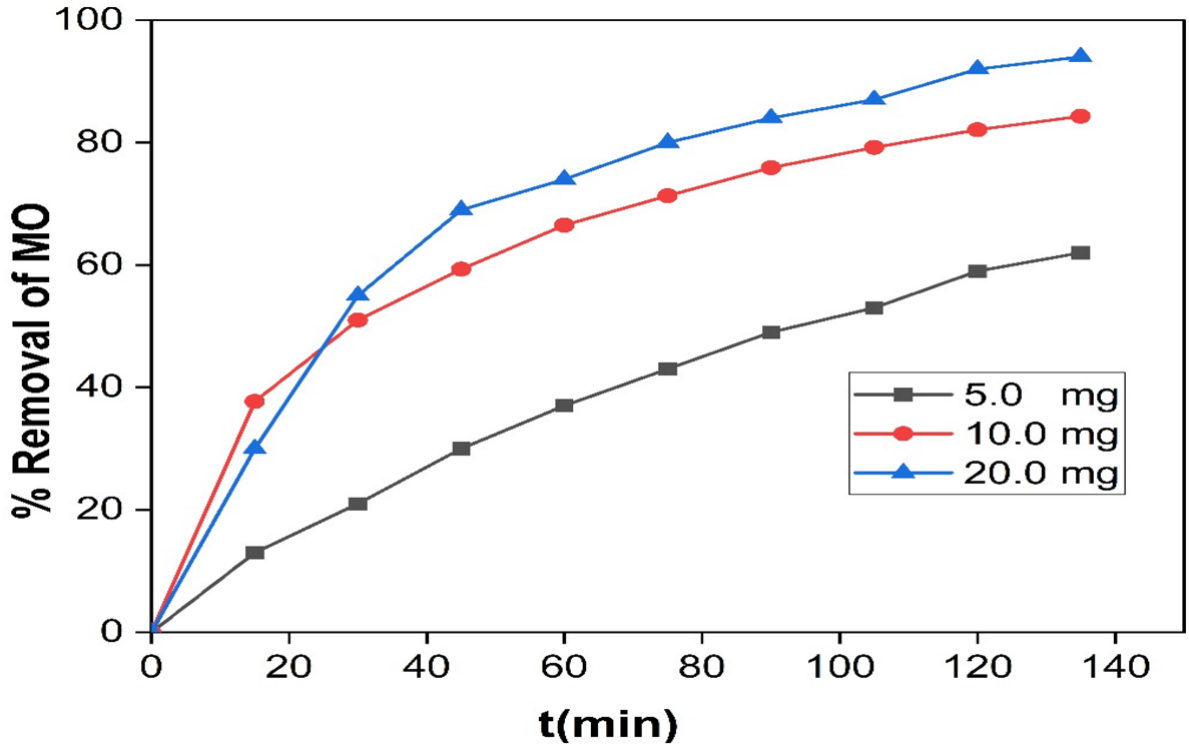
Effect of the photocatalyst dose on the Removal efficiency of MO (50 ml MO solution (10 mg/l), Temp. = 25 °C and pH 3.0).

The influence of a nanocomposite dose on MO removal efficiency under visible-light was investigated via different photocatalyst doses between 5 and 20 mg against a fixed concentration of MO (10 mg/l) (Fig. 11).

The results indicated an increase in removal efficiency with the increase in photocatalyst dose from (5–20 mg). It can be concluded that the maximum removal efficiency of MO was reached 95% at 20 mg of Ag/Mn_2_O_3_ NPs. The increase in removal efficiency with catalyst amount could be attributed to the increase in the available active area or active sites of the photocatalyst to volume ratio of MO solution^37^.

#### Kinetic studies

The MO removal rate was computed using the following equation:

3 $$-ln Ct/C0 = - Kt$$

**Figure 12:**
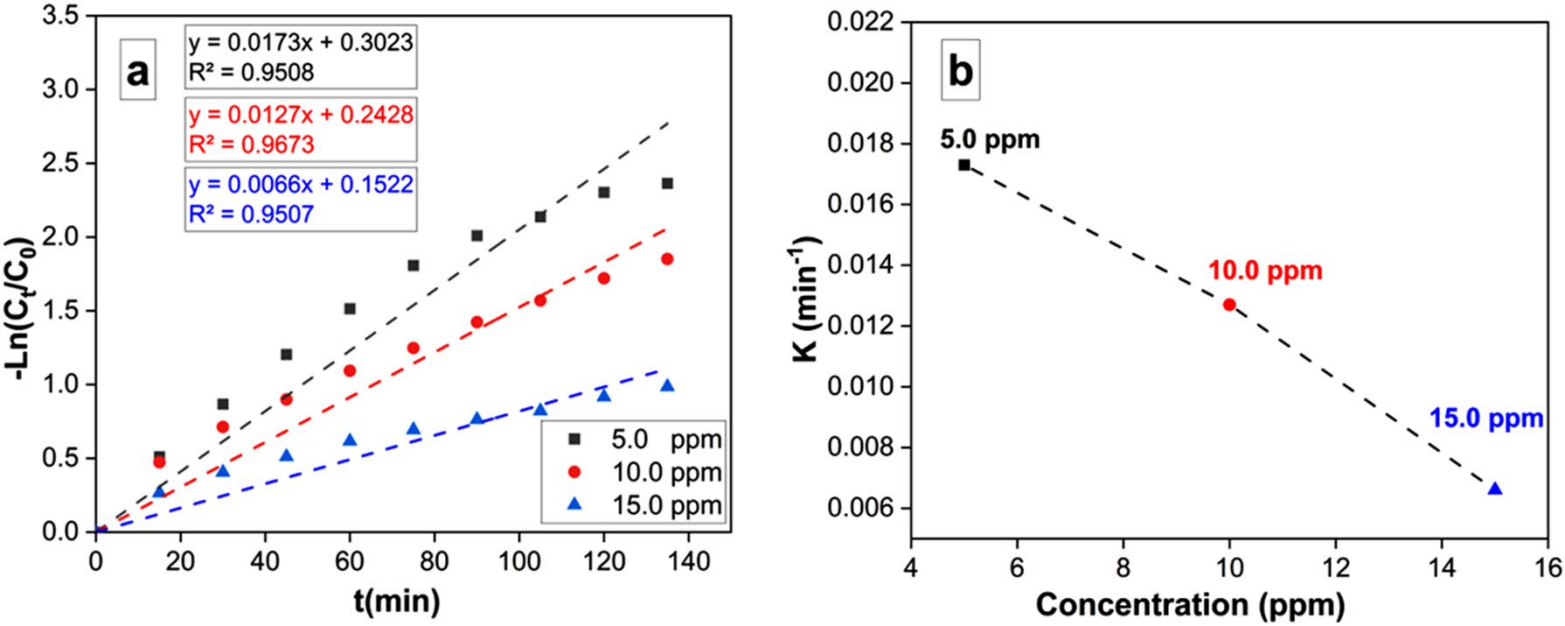
A linear fit, pseudo-first-order model data are reported in kinetic form for MO degradation under Visible-light irradiation with beginning MO concentrations of 5, 10, and 15 ppm.

Here, Ct and Co denote for the remaining and initial concentrations of MO, respectively. The removal time is represented by t, and k represents the removal rate constant. The relationship between -ln (C_t_/C_o_) and t is represented in Fig. 12a. These findings indicated that the removal reaction kinetics adhered to pseudo-first-order rate. An augmentation in the MO initial concentration resulted in an increase in the apparent pseudo-first-order rate constants (Fig. 12b).

This dependency of reaction rate constants on MO concentration aligns well with previously reported literature^38^. Pseudo-first-order kinetics could be a useful tool for initial analysis, it simplifies the photocatalytic degradation process. It assumes the reaction rate solely depends on the concentration of the pollutant (MO). In reality, other factors like light intensity, catalyst surface area, and mass transfer limitations can significantly impact the degradation rate. Photocatalysis involves multiple steps and interactions between light, catalyst, and pollutants. However, the pseudo-first-order model is a valuable tool for initial data analysis. In this study, pseudo-second-order kinetics might be a better fit because the adsorption of the pollutant onto the catalyst plays a significant role.

#### Mechanism of MO photocatalysis

The potential mechanism could be correlated to photodegradation mechanism, which is influenced by variations in pH values. Such mechanism encompasses the attack of hydroxyl radicals, with subsequent oxidation through the positive holes in the valence band, and reduction by the electrons in the conduction band. It is postulated that photocatalytic degradation is likely to occur due to the generation of electron–hole pairs on the surface of the employed photocatalyst induced by visible irradiation^39,40^.

The oxidative potential of these holes may either react with -OH groups to form hydroxyl radicals or oxidize the reactive MO to generate a degradation product. The reactions involved in MO photocatalytic degradation can be summarized as follows (4–7).

4 $$ {\text{Ag/Mn}}_{{2}} {\text{O}}_{{3}} {\text{NPs + hv}} \to {\text{Ag/Mn}}_{{2}} {\text{O}}_{{3}} {\text{NPs (e}}^{ - }_{{{\text{CB}}}} {\text{ + h}}^{ + }_{{{\text{VB}}}} {)} $$

5 $$ {\text{h}}^{ + }_{{{\text{VB}}}} {\text{ + Ag/Mn}}_{{2}} {\text{O}}_{{3}} {\text{NPs}} \to {\text{Ag/Mn}}_{{2}} {\text{O}}_{{3}} {\text{NPs}}^{ + } \;\left( {\text{Oxidation of the compound}} \right) $$

Or

6 $$ {\text{h}}^{ + }_{{{\text{VB}}}} {\text{ + OH}}^{ - } \to {\text{OH}} $$

7 $$ {\text{OH}}^{.} {\text{ + MO}} \to \left( {\text{Degradation products}} \right) $$

**Figure 13:**
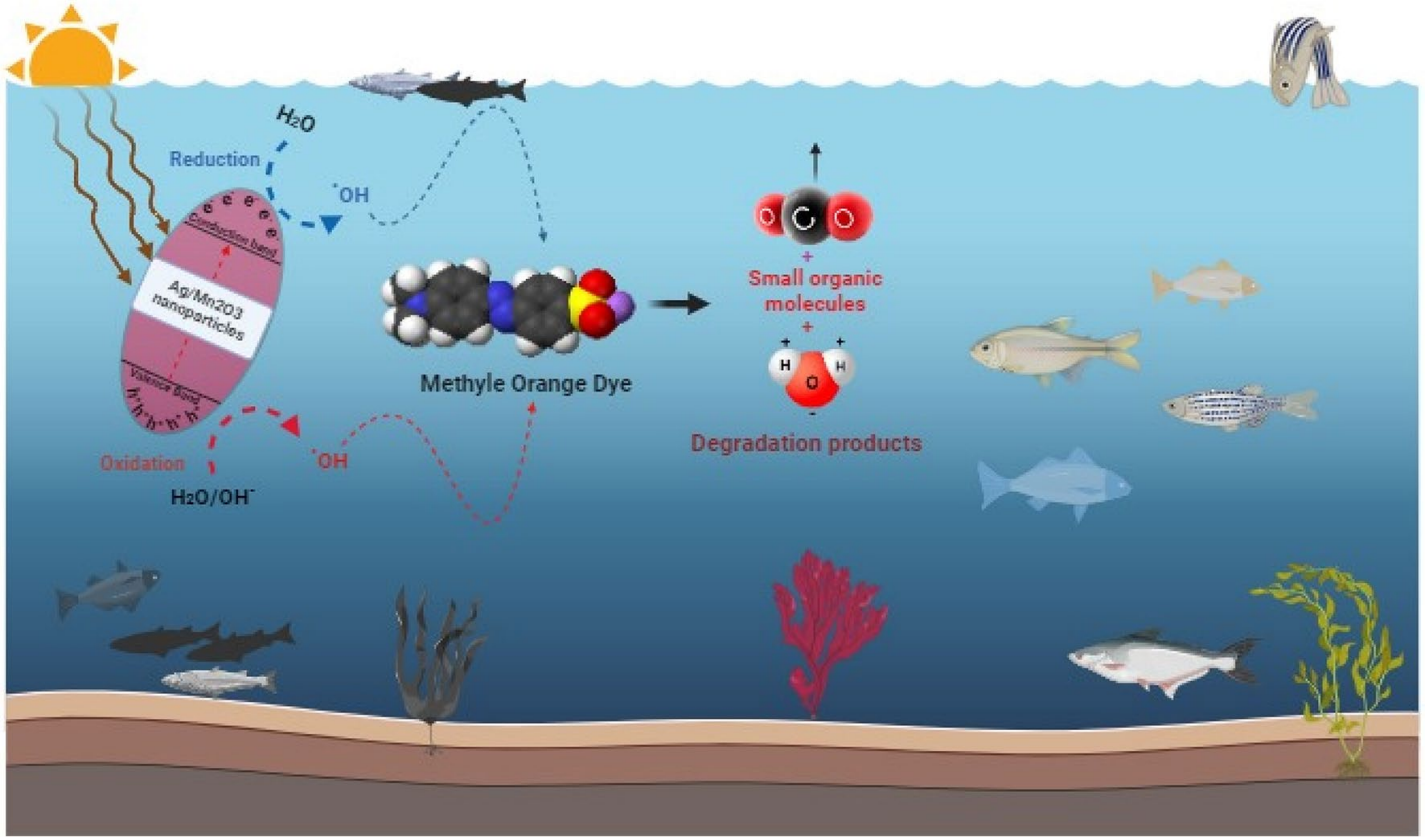
The possible photocatalytic reaction mechanism for MO photodegradation by Ag/Mn_2_O_3_ NPs.

The suggested mechanism delineating the interaction between the prepared Ag/Mn_2_O_3_ NPs and MO is illustrated in Fig. 13. When Ag/Mn_2_O_3_ NPs are subjected to visible irradiation, the excitation process could generate charge carriers; that could initiate redox reactions. Consequently, the resultant free radicals, including OH· and O_2_·^−^ could participate in the degradation of MO, with the formation of smaller organic compounds. It is noteworthy that, as of the current stage, there have been no published reports regarding the degradation mechanism of MO.

### Antimicrobial and antibiofilm activities of synthesized nanoparticles

#### Antimicrobial activity of synthesized Ag/Mn_2_O_3_ nanocatalyst

**Figure 14:**
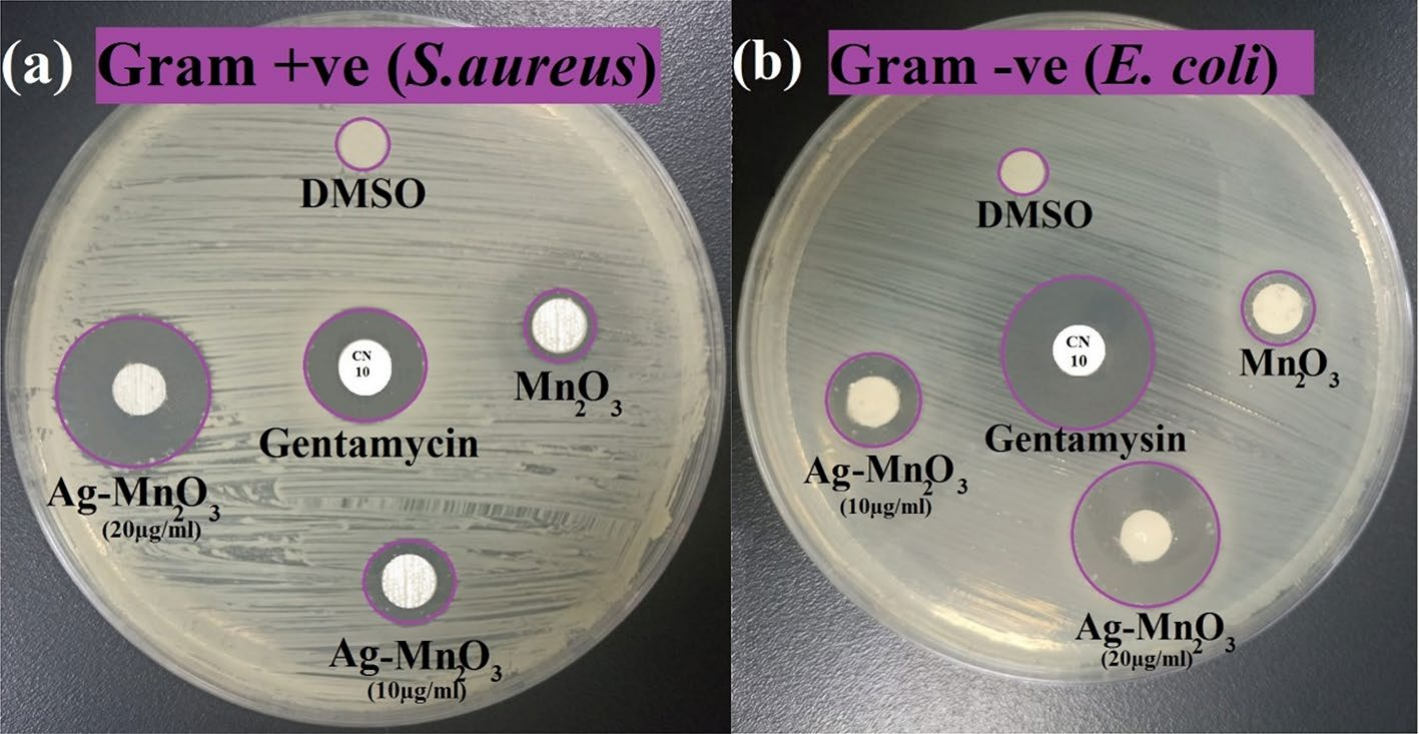
Antimicrobial activity of the synthesized NPs; Mn_2_O_3_, Ag/Mn_2_O_3_ (10 and 20 µg/ml), measured as ZOI (mm) against (**a**) gram positive S. aureus, (**b**) gram negative E. coli.

The in-vitro ZOI result verified that Ag/Mn_2_O_3_ NPs (20µg/ml) exhibited enhanced antibacterial activity against *S. aureus* (19 mm ZOI), and *E. coli* (22 mm ZOI) respectively (Fig. 14).

The results demonstrated that the synthesized Ag/Mn_2_O_3_ NPs appeared more active towards the Gram-negative compared to that of Gram-positive bacteriological species. This may be due to structural and compositional variations between Gram-negative and Gram-positive bacterial cell walls^7^. Gram-positive bacteria have a dense coating of peptidoglycan to which teichuronic, and teichoic acids are covalently bound. However, Gram-negative bacteria have a thin coating of peptidoglycan with an exterior coating of negatively charged lipopolysaccharides^8^. Due to such reasons, the synthesized Ag/Mn_2_O_3_ NPs could expose significant inhibitory effect on Gram-negative than on Gram-positive bacterial species (Table 4).

**Table 4:** Antimicrobial activities of Ag/Mn_2_O_3_, against gram-positive and gram-negative bacteria measured as ZOI (mm), MIC (µg/ml) and antibiofilm activity of Ag/Mn_2_O_3_.

Bacterial Strains	ZOI of Mn_2_O_3_ (10.0 µg/ml) (mm)	ZOI of Ag/Mn_2_O_3_ (10.0 µg/ml) (mm)	ZOI of Ag/Mn_2_O_3_ (20.0 µg/ml) (mm)	MIC of Ag/Mn_2_O_3_ NPs (µg/ml)	CN	Antibiofilm (%)
*S. aureus*	7.0	9.0	19.0	2.50	14.0	89.5
*E. coli*	8.0	11.0	22.0	1.250	22.0	95.4

#### Antibiofilm activity of Ag/Mn_2_O_3_ nanocatalyst

**Figure 15:**
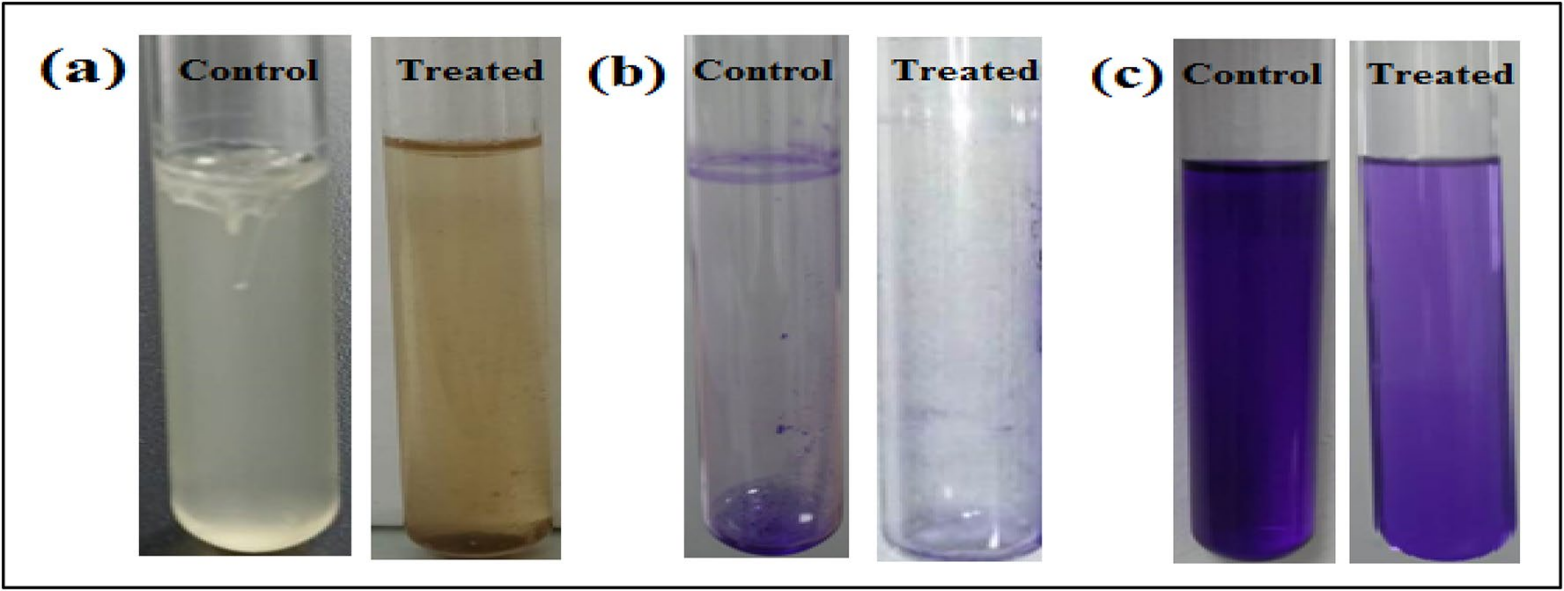
Antibiofilm activity of Ag/Mn_2_O_3_ NPs using the test tube method against E. coli. The steps were reported as follows. (**a**) Growth of the bacterial cells and biofilm formation (rings) without treatment with the synthesized Ag/Mn_2_O_3_ NPs and the inhibition of bacterial growth after treatment with Ag/Mn_2_O_3_ NPs. (**b**) Staining of the adherent bacterial cells with crystal violet. (**c**) Removing and dissolving the adherent bacterial and yeast cells by ethanol for determination of semi-quantitative biofilm inhibition (%) (Table 4).

Biofilm generation has been recognized in several exopolysaccharide-forming microbes^41,42^. Biofilm production by common pathogenic bacteria microorganisms in the absence and presence of Ag/Mn_2_O_3_ NPs was assessed using a test tube method^43^. Figure 15a demonstrates the antibiofilm activity of Ag/Mn_2_O_3_ NPs against *E. coli* bacteria (as a model for susceptible bacteria). *E. coli* inoculated in the absence of Ag/Mn_2_O_3_ nanocomposite exhibited a thick whitish-yellow mat throughout the air–liquid interface. This mat was totally adhered to the wall of the test tubes and appeared as a blue ring after CV staining as displayed in Fig. 15b. A blue suspension was developed after dissolving the CV-stained ring with absolute ethanol, as displayed in Fig. 15c.

**Figure 16:**
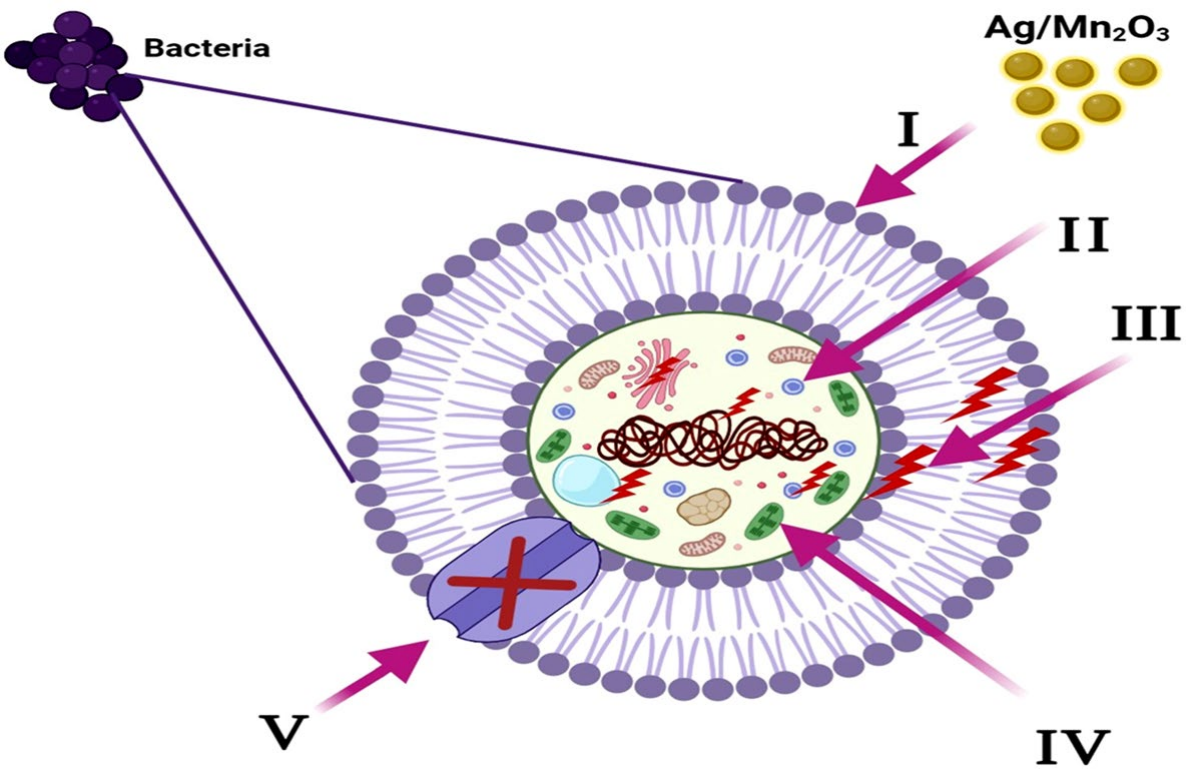
Schematic representation of the four main pathways underlying the antibacterial potential of the prepared Ag/Mn_2_O_3_ NPs: (I) The Ag/Mn_2_O_3_ NPs adhere to and wrap the microbial cell surface causing membrane damage and altered transport activity. (II) The prepared Ag/Mn_2_O_3_ NPs penetrate the microbial cells and interact with cellular organelles and biomolecules (such as plasmid DNA, ribosomes, chromosomal DNA, and mesosomes), affecting the respective cellular machinery. (III) The prepared Ag/Mn_2_O_3_ NPs create and increase ROS, leading to cell damage. (IV) Ag/Mn_2_O_3_ NPs modulate the cellular signal system and causing cell death. (V) Finally, Ag/Mn_2_O_3_ NPs nanocomposite blocks the ion transport from and to the microbial cells.

On the other hand, *E. coli*-inoculated test tubes that were treated with Ag/Mn_2_O_3_ NPs (10.0 µg ml^−1^), exhibited a suppressed effect where the development of bacterial rings was limited. In addition, the blue color representing CV-stained adherent bacterial cells was light and, after CV dissolution by ethanol, slightly blue color was observed (Fig. 15c). The optical density (at 570.0 nm) was determined after separating CV-stained bacterial biofilm with ethanol. Table 3 demonstrates the inhibition percentage of the biofilms formed by the tested bacteria. The highest inhibition percentage was observed against *E. coli* (95.5%; Fig. 16 and Table 4) followed by *S. aureus* (89.5%; Table 4) after treatment with 10.0 µg ml^-1^ of Ag/Mn_2_O_3_ NPs. The synthesized Ag/Mn_2_O_3_ nanocatalyst was adopted to restrain the development of biofilm at its adhesion step (identified as the primary step). The variance in the percentage inhibition could be attributed to various circumstances, such as antimicrobial action, biosorption (because of the large surface area of the synthesized NPs), physical characteristics (particle size of the nanocomposite), invasion capabilities, and different chemical features that could control the interaction of the nanocomposite and the biofilms.

#### Mechanism of antimicrobial effect of the prepared Ag/Mn_2_O_3_ NPs

The antimicrobial mechanism of the prepared Ag/Mn_2_O_3_ NPs was proposed. The prepared Ag*/*Mn_2_O_3_ NPs begin their activity by wrapping and adhering at the exterior surface of the microbial cells, causing membrane destruction and changing the transport potential^44^. Then, the distribution of the NPs inside the microbial cell divides all intracellular structures, including plasmid, DNA, and other essential organelles^45^. Ultimately, cellular toxicity happens due to the oxidative stress created by the generation of ROS^46^. Finally, nanocomposite blocks the ion transport from and to the microbial cells (Fig. 16).

## Conclusion

Manganese oxide nanorods were prepared by hydrothermal processing. The developed Mn_2_O_3_ NPs were doped with silver. The photocatalytic efficiency of the prepared Ag-Mn_2_O_3_ was assessed against methyl orange dye. Degradation efficiencies Mn_2_O_3_ and Ag/Mn_2_O_3_ on methyl orange (MO) dye was found to be 53% and 85% under visible spectrum. Silver dopant was reported to decrease the binding energy of valence electrons; this action could support electon-hole pair generation under visible spectrum and could promote catalytic performance. Ag/Mn_2_O_3_ NPs demonstrated most effective performance (95% removal efficiency) at pH 3; this could be ascribed to the electrostatic attraction between positively charged catalyst and the negatively charged MO. Ag/Mn_2_O_3_ demonstrated enhanced antibacterial activity against *S. aureus* (19 mm ZOI), and *E. coli* (22 mm ZOI) respectively; the developed nanocomposite demonstrated advanced anti-film activity with inhibition percentage of 95.5% against *E. coli* followed by 89.5% against *S. aureus*.

### Supplementary Information

Supplementary Figures.

## Data Availability

The data used to support the findings of this study are available from the corresponding author upon request.
