# Supplementary material for: Facile synthesis of silver doped manganese oxide nanocomposite with superior photocatalytic and antimicrobial activity under visible spectrum
Source: Sci Rep. 2024 Jul 8;14:15658. doi: 10.1038/s41598-024-65749-z (PMC11231171; doi:10.1038/s41598-024-65749-z)
Supplement: Supplementary file 1 — Supplementary Figures. [file 41598_2024_65749_MOESM1_ESM.docx]

**Supplementary Figures**

**(A)**

H_2_O

400 ^0^C and 240 bars


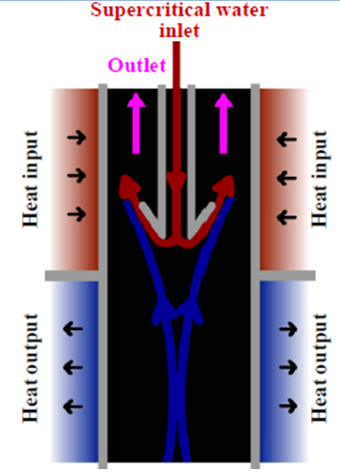

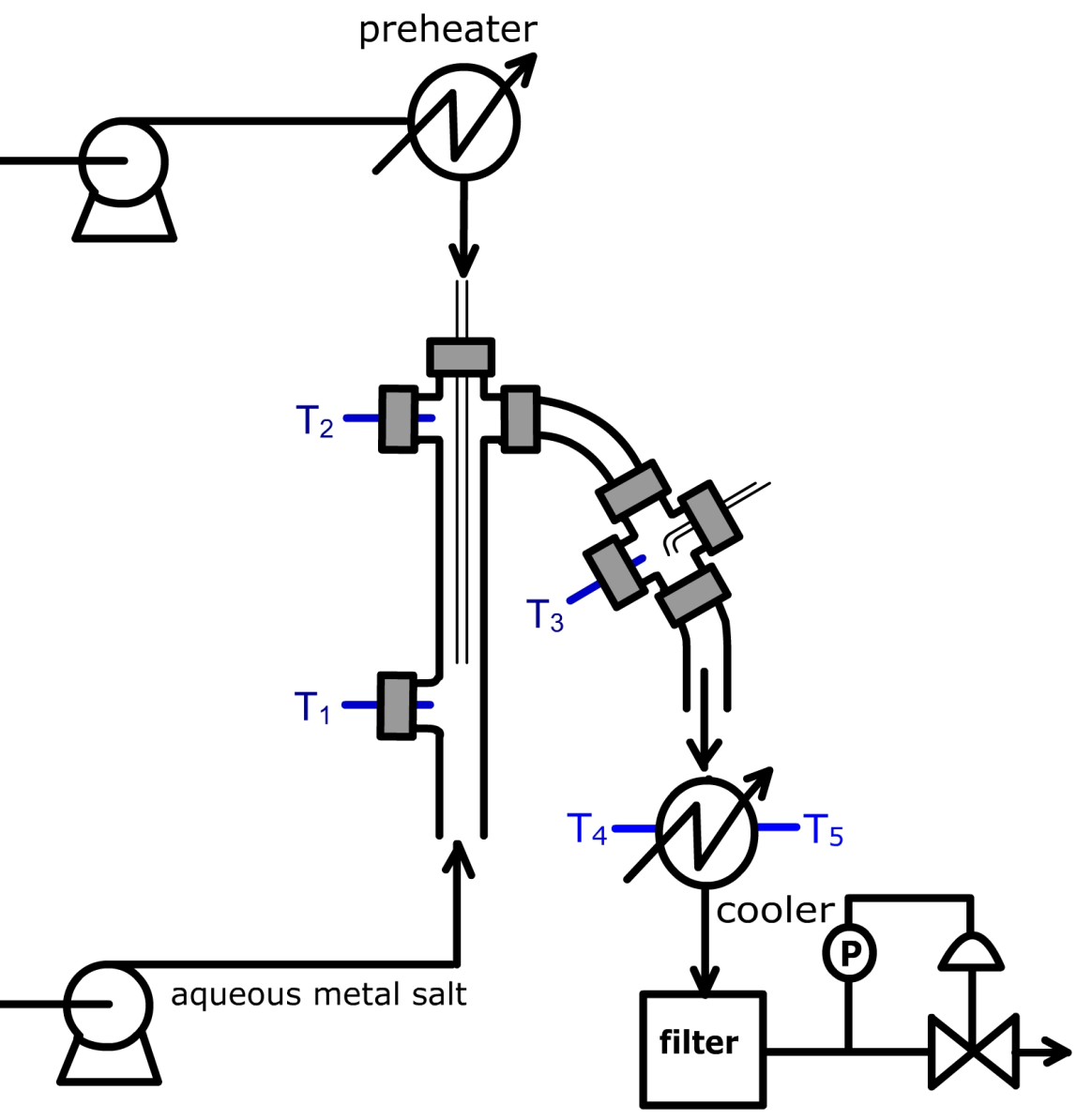


**(B)**

0.05 M Manganese nitrate

25 ^0^C, 240 bars

**Pump 1**

**Pump 2**

**Fig. S1: Schematic for Continuous hydrothermal synthesis of Mn_2_O_3_ nanorods**

**
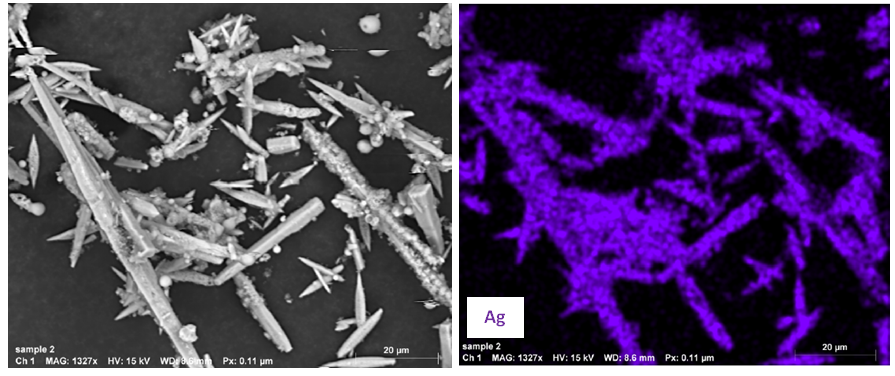
**

**Fig. S2: Elemental mapping using EDAX detector of Ag/Mn_2_O_3_**
